# Kinin B_1_ and B_2_ Receptors Contribute to Cisplatin-Induced Painful Peripheral Neuropathy in Male Mice

**DOI:** 10.3390/pharmaceutics15030852

**Published:** 2023-03-06

**Authors:** Gabriela Becker, Maria Fernanda Pessano Fialho, Indiara Brusco, Sara Marchesan Oliveira

**Affiliations:** Graduate Program in Biological Sciences: Biochemical Toxicology, Center of Natural and Exact Sciences, Federal University of Santa Maria, Camobi, Santa Maria 97105-900, RS, Brazil

**Keywords:** bradykinin, neuropathic pain, chemotherapy, allodynia, CIPN

## Abstract

Cisplatin is the preferential chemotherapeutic drug for highly prevalent solid tumours. However, its clinical efficacy is frequently limited due to neurotoxic effects such as peripheral neuropathy. Chemotherapy-induced peripheral neuropathy is a dose-dependent adverse condition that negatively impacts quality of life, and it may determine dosage limitations or even cancer treatment cessation. Thus, it is urgently necessary to identify pathophysiological mechanisms underlying these painful symptoms. As kinins and their B_1_ and B_2_ receptors contribute to the development of chronic painful conditions, including those induced by chemotherapy, the contribution of these receptors to cisplatin-induced peripheral neuropathy was evaluated via pharmacological antagonism and genetic manipulation in male Swiss mice. Cisplatin causes painful symptoms and impaired working and spatial memory. Kinin B_1_ (DALBK) and B_2_ (Icatibant) receptor antagonists attenuated some painful parameters. Local administration of kinin B_1_ and B_2_ receptor agonists (in sub-nociceptive doses) intensified the cisplatin-induced mechanical nociception attenuated by DALBK and Icatibant, respectively. In addition, antisense oligonucleotides to kinin B_1_ and B_2_ receptors reduced cisplatin-induced mechanical allodynia. Thus, kinin B_1_ and B_2_ receptors appear to be potential targets for the treatment of cisplatin-induced painful symptoms and may improve patients’ adherence to treatment and their quality of life.

## 1. Introduction

Cancer incidence is increasing yearly, with an expected expansion rate of approximately 50% by 2040 [1]. Concomitantly, remarkable improvements in the survival rates of cancer patients have been observed due to advances in early detection and available treatments [2,3,4]. With the increasing number of cancer survivors, more attention must be given to the potential risk of developing severe adverse effects associated with therapy, such as chemotherapy-induced peripheral neuropathy (CIPN) [3,5,6].

CIPN is the most frequent and potentially permanent neurological complication of cancer treatment [5,7,8]. Platinum-based chemotherapeutics, such as cisplatin, are associated with a high incidence of CIPN and may affect up to 85% of treated patients [5,7,8,9,10,11].

Cisplatin treats highly prevalent tumours such as those of bladder, ovarian, testicular, lung, and head and neck cancers, as well as sarcomas [12]. However, cisplatin accumulation in the dorsal root ganglia neurons causes neuronal dysfunction and apoptosis, often resulting in irreversible changes in the peripheral nervous system, leading to peripheral neuropathy [7,9,13]. The incidence and severity of cisplatin-induced peripheral neuropathy are dose-dependent, and the symptoms appear during or after treatment [6,7,8]. Consequently, this condition can lead to dose reduction and treatment discontinuation, affecting overall patient survival [6,8,9,14].

Clinically, CIPN sensory symptoms are predominant in patients and can persist for months after completion of chemotherapy [7,8,14,15]. They usually develop first in the feet and hands; however, prolonged treatment may aggravate the signs and symptoms and extend to more proximal limb areas [6,8,9,16]. Patient symptoms manifest as spontaneous or evoked abnormal sensations such as paraesthesia, dysesthesias, numbness, and tingling. In addition, neuropathic-like painful sensations are frequently reported, such as mechanical or thermal allodynia or hyperalgesia, burning pain, and shooting or electric shock-like pain [6,8,16]. An essential aspect of platinum-based CIPN is the “coasting” phenomenon, whereby the signs and symptoms may worsen months after the discontinuation of chemotherapy [9,12,16].

Currently, there are no preventive strategies to attenuate this painful condition, and treatment is limited and commonly ineffective in many patients [14,15]. Although various pharmacologic agents have been evaluated for the treatment of CIPN, only duloxetine has been moderately recommended by the American Society of Clinical Oncology [14,15]. Given the limited treatment options for CIPN, it is necessary to identify efficacious and well-tolerated novel pharmacological strategies for CIPN symptoms without affecting cancer treatment regimens.

In this sense, kinin B_1_ and B_2_ receptors activated by kinins have attracted attention due to their involvement in nociceptive processes and different painful conditions [17,18,19,20,21,22,23]. Bradykinin (Bk) and kallidin target the B_2_ receptor, while the B_1_ receptor has a higher affinity for the active metabolites of kinins, namely des-Arg^9^-Bk (DABk) and des-Arg^10^-kallidin. Nociceptive neurons express kinin B_1_ and B_2_ receptors [21,24,25,26], which, when activated, cause painful nociceptive responses in humans and experimental animals [20,27,28,29]. Kinin B_1_ and B_2_ receptors mediate the acute and chronic pain induced by various pain models [17,18,20,21,22,23,30], including chemotherapy drugs such as paclitaxel and vincristine [27,31,32,33]. Furthermore, kinin B_1_ and B_2_ receptors are involved in cisplatin-induced nephrotoxicity since the pharmacological blockade and knockout animals of kinin receptors attenuate acute kidney injury [34,35].

Due to the significant implications for cancer survivors, it is important to gain an understanding of the principal pathophysiological mechanisms involved in chemotherapy-induced painful symptoms to aid in the search for potential therapies to prevent or minimize these pain symptoms. In this respect, using a model of cisplatin-induced painful neuropathy in mice, we evaluated the involvement of the kinin B_1_ and B_2_ receptors in the pain symptoms induced by cisplatin.

## 2. Materials and Methods

### 2.1. Drugs and Reagents

Cisplatin (cis-diamminedichloridoplatinum II, C-Platin^®^; Blau, SP, Brazil), bradykinin (Bk; kinin B_2_ receptor agonist), Icatibant (peptide kinin B_2_ receptor antagonist), des-Arg^9^-bradykinin (DABk; kinin B_1_ receptor agonist), and des-Arg^9^-[Leu^8^]-bradykinin (DALBK; peptide kinin B_1_ receptor antagonist) were purchased from Sigma Chemical Company (St. Louis, MO, USA). FR173657 (non-peptide kinin B_2_ receptor antagonist) and SSR240612 (non-peptide kinin B_1_ receptor antagonist) were obtained from Sanofi-Aventis (Berlin, Germany). Antisense oligonucleotides targeting the kinin B_1_ receptor (5′-AGG TTC CTG TGG ATG GCG TCCC-3′), kinin B_2_ receptor (5′-AGA ATT CTG TTC ACT GTT TCT TCC CTG-3′), and nonsense oligonucleotides (5′-GGT GGA T TTG AGG ATT TCG GC-3′) were acquired from GenOne Biotechnologies (Rio de Janeiro, Brazil). Cisplatin and antagonists were prepared in a saline solution (0.9%). Phosphate-buffered saline (PBS; 10 mM) was used to dilute reagents administered via the intraplantar route (kinin B_1_ and B_2_ receptor agonists). The control groups (vehicles) received the vehicles where the treatments were solubilized. All the intraperitoneal treatments were administered in mice in a volume of 10 mL/kg, while intraplantar injections were administered in a volume of 20 µL.

### 2.2. Animals

The experiments were conducted using adult male Swiss mice (25–30 g; 4–5 weeks of age) produced and provided by the Federal University of Santa Maria. The animals were maintained in a temperature-controlled room (22 ± 1 °C) under a 12 h light/12 h dark cycle with free access to food and water. Experimental protocols were performed with the approval of the Institutional Animal Care and Use Committee of the Federal University of Santa Maria (approval processes #7152261119/2020 and #6380261021/2022). Experimental protocols were conducted according to the guidelines for investigation of experimental pain in conscious animals [36,37], the Animal Research: Reporting in vivo Experiments ARRIVE guidelines [38], and national and international legislation (guidelines of the Brazilian Council of Animal Experimentation Control (CONCEA) and the U.S. Public Health Service’s Policy on Humane Care and Use of Laboratory Animals (PHS policy)). The number of animals and the intensities of noxious stimuli used were the minimum necessary to demonstrate the consistent effects of the treatments. Behavioural experiments were conducted in a quiet, temperature-controlled room (20 °C to 22 °C) between 9 a.m. and 5 p.m. and were performed by investigators blinded to the treatment conditions. The group size for each experiment was based on studies with protocols similar to ours [27,39,40], which were confirmed by power calculations (G*Power version 3.1.9.7).

### 2.3. Cisplatin-Induced Peripheral Neuropathy Model

**Figure 1:**
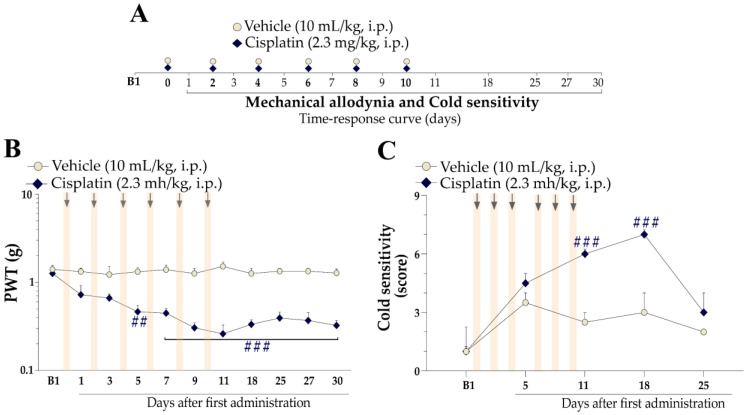
Characterization of cisplatin-induced painful peripheral neuropathy. (**A**) Male Swiss mice were treated intraperitoneally (i.p.) with cisplatin (2.3 mg/kg) or its vehicle (10 mL/kg) from day 0 onwards every other day (days 0, 2, 4, 6, 8, and 10) for a total of 10 days to induce the peripheral neuropathy experimental model. The mice’s mechanical PWT was assessed between the treatment days until 30 days after the first cisplatin administration (**B**). Cold sensitivity was evaluated on the 5th, 11th, 18th, and 25th days after the first cisplatin administration (**C**). B1 denotes baseline values measured before the first cisplatin or vehicle dose. Data are expressed as the mean + SEM (n = 6/group) and were analysed by two-way ANOVA followed by Bonferroni post hoc test. ^##^ *p* < 0.01 and ^###^ *p* < 0.001 when compared to the vehicle group. The arrows represent the days of cisplatin or vehicle administration. PWT: paw withdrawal threshold.

To establish the cisplatin-induced peripheral neuropathy model, the mice were treated with intraperitoneal (i.p.) injections of cisplatin at a dose of 2.3 mg/kg administered every 48 h for 10 days (days 0, 2, 4, 6, 8, and 10), totalling 6 doses of cisplatin [41,42]. The control group received the vehicle (10 mL/kg, i.p.; saline solution [0.9%]), employing the same administration schedule. Mannitol (125 mg/kg, intraperitoneal) was administered 1 h before cisplatin to avoid renal toxicity [43]. After the first cisplatin or vehicle injection, the animals were subjected to behavioural assessments. The experimental design is represented in Figure 1A.

### 2.4. Study Design for Behavioural Assessment

Mechanical paw withdrawal threshold (PWT) and cold sensitivity were evaluated before the cisplatin administration protocol (baseline, B1). Next, the mice received vehicle (control group; 10 mL/kg, i.p.) or cisplatin (2.3 mg/kg, i.p.). The PWT was continuously assessed 24 h after each cisplatin or vehicle administration up to 30 days after the first administration [following the protocol described below (2.6.1 Mechanical allodynia assessment)]. Cold sensitivity was evaluated on days 5, 11, 18, and 25 after the first cisplatin or vehicle administration [following the protocol described below (2.6.2 Cold sensitivity)]. The locomotor activity of the animals was evaluated in an open field on the 11th and 30th days after the first administration of cisplatin or vehicle [following the protocol described below (2.7.2 Locomotor Activity)].

Spontaneous pain was assessed by the voluntary wheel activity and nesting behaviour [following the protocols described below (2.6.3 Voluntary wheel activity and 2.6.4 Nesting behaviour test)]. Anxiety and depressive-like behaviours, as well as cognitive function, were assessed by thigmotaxis behaviour and a forced swimming test, as well as a novel object/place recognition test, respectively [following the protocols described below (2.6.5 Thigmotaxis behaviour, 2.6.6 Forced swimming test, and 2.6.7 Novel object place recognition test)].

### 2.5. Study Design for the Assessment of Kinin B_1_ and B_2_ Receptor Involvement in Cisplatin-Induced Painful Behaviours

A therapeutic and preventive protocol using kinin B_1_ and B_2_ receptor antagonists was performed to evaluate their contribution to the mechanical and cold painful hypersensitivity induced by cisplatin. Agonists and antisense oligonucleotides for the kinin B_1_ and B_2_ receptors were also used.

#### 2.5.1. Therapeutic Protocol

The therapeutic protocol was designed to evaluate the effect of kinin B_1_ and B_2_ receptor antagonists in mice with mechanical and cold allodynia previously established by cisplatin. The mechanical PWT and cold sensitivity of animals were measured before the cisplatin (2.3 mg/kg, i.p.) administrations (baseline values; B1) and 24 h after the last injection (11th day) (baseline values; B2). Next, the mice received a single intraperitoneal (i.p.) administration of the peptide kinin B_1_ or B_2_ receptor antagonist, i.e., DALBK (150 nmol/kg, i.p.) or Icatibant (100 nmol/kg, i.p.), respectively. The mechanical PWT and cold sensitivity were evaluated at different time points following the treatments (from 0.5 h up to 4 h). The mechanical PWT and cold sensitivity were also evaluated after treatments (from 0.5 to 24 h) with non-peptide kinin B_1_ (SSR240612, 150 nmol/kg, i.p.) or B_2_ (FR173657; 100 nmol/kg, i.p.) receptor antagonists. The experimental design is represented in Figure 3A.

#### 2.5.2. Preventive Protocol

The preventive protocol was delineated to evaluate the capacity of the kinin B_1_ and B_2_ receptor antagonists to prevent the development of cisplatin-induced mechanical allodynia and cold sensitivity. Mechanical PWT and cold sensitivity were measured before cisplatin and treatments (baseline values; B1). After baseline measurements, the mice were treated concomitantly every 48 h for 10 days with cisplatin (2.3 mg/kg, i.p.) + vehicle (10 mL/kg, i.p.), cisplatin (2.3 mg/kg, i.p.) + kinin B_1_ receptor antagonist (DALBK, 150 nmol/kg, i.p.), or cisplatin (2.3 mg/kg, i.p.) + kinin B_2_ receptor antagonist (Icatibant, 100 nmol/kg, i.p.). Mechanical PWT and cold sensitivity were assessed 24 h after each administration up to 14 days after the first cisplatin administration. The experimental design is represented in Figure 4A.

#### 2.5.3. Effects of Sub-Nociceptive Doses of Kinin B_1_ and B_2_ Receptor Agonists on Mechanical Allodynia in Mice with Cisplatin-Induced Peripheral Neuropathy

We examined whether low doses of kinin B_1_ and B_2_ receptor agonists could enhance the mechanical nociception of cisplatin-treated mice. The animals were previously treated with cisplatin (2.3 mg/kg, i.p.) or vehicle (10 mL/kg, i.p.). Twenty-four hours after the last cisplatin administration (11th day), the animals received an intraplantar (i.pl.) injection of DABk (3 nmol/paw, kinin B_1_ receptor agonist) or Bk (1 nmol/paw, kinin B_2_ receptor agonist), all in sub-nociceptive doses, or their vehicles (20 µL PBS/paw, i.pl.), and the mechanical PWT was evaluated again from 0.5 up to 3 h and from 0.5 up to 2 h after the agonist injections, respectively. The experimental design is represented in Figure 5A.

To confirm the involvement of kinin B_1_ and B_2_ receptors in mechanical allodynia, the mice were treated with DALBK (150 nmol/kg, i.p.) or Icatibant (100 nmol/kg, i.p.) 24 h after the last cisplatin dose (11th day). After 0.5 h, the same animals were treated with sub-nociceptive doses of the respective kinin B_1_ and B_2_ receptor agonists—DABk or Bk—by the intraplantar route. Next, the PWT was assessed until treatments with the antagonists showed an effect. The experimental design is represented in Figure 6A.

#### 2.5.4. Effects of Antisense Oligonucleotides for Kinin B_1_ and B_2_ Receptors on Cisplatin-Induced Mechanical and Cold Sensitivity in Mice

To confirm the contribution of kinin B_1_ and B_2_ receptors to cisplatin-induced painful behaviours, mice were treated with intrathecal injections of an antisense oligonucleotide for kinin B_1_ and B_2_ receptors or their control. First, baseline (B1) mechanical PWT and cold sensitivity were measured. Then, the animals were treated with 6 doses of cisplatin (2.3 mg/kg, i.p.). Twenty-four h after the last cisplatin administration (11th day), mechanical and cold sensitivity were evaluated again (baseline values; B2). Next, the animals were treated intrathecally (5 μL; between L5 and L6) twice a day (12/12 h) for three consecutive days with antisense oligonucleotides targeting the kinin B_1_ receptor (antisense B_1_; 5 μg/site) and the kinin B_2_ receptor (antisense B_2_; 5 μg/site) or the control oligonucleotide (nonsense, 5 μL/site) [18]. On the fourth day, the animals received one last administration of antisense or nonsense oligonucleotides 1 h before evaluating mechanical PWT and cold sensitivity. The experimental design is represented in Figure 7A.

### 2.6. Behavioural Experiments

#### 2.6.1. Mechanical Allodynia Assessment

Mechanical allodynia was assessed using flexible nylon filaments (von Frey) of increasing stiffness (0.02–10 g) by the up-and-down method [44,45]. The mechanical PWT response, expressed in grams (g), was calculated from the resulting scores using von Frey filaments, according to previous studies [23,27,45]. Mechanical allodynia was considered a decrease in the PWT compared with the baseline values (B1) before cisplatin administration.

#### 2.6.2. Cold Sensitivity

Cold sensitivity was assessed with the acetone drop method as previously described [23,46]. The mice were placed on a wire mesh floor, and a drop of acetone (20 µL) was applied three times on the plantar surface of the right hind paw. The behavioural response was analysed for 30 s and recorded in scores. The scores were: 0 = no response; 1 = quick withdrawal, flick, or stamp the paw; 2 = prolonged withdrawal or repeated paw flicking; and 3 = repeated paw flicking with licking directed at the ventral side of the paw. The sum of the three scores was used for data analysis. Cold sensitivity was considered an increase in the scores compared with the baseline values before cisplatin administration.

#### 2.6.3. Voluntary Wheel Activity

The running activity is a simple, observer-independent objective measure and provides a measure of spontaneous activity in a known environment, potentially reflecting whether the activity is painful [47]. The voluntary wheel activity was assessed in polycarbonate cages with free access to stainless steel activity wheels (Wheel activity EP 172—Insight, Ribeirão Preto, SP, Brazil). The wheel could be turned in either direction. The wheels were connected to a digit counter that automatically recorded the number of turns. First, mice were habituated in individual activity cages for three sessions over at least three days. The distance travelled by each animal on the wheel during each 1 h evaluation session was obtained by multiplying the number of turns by the wheel diameter (30 cm). The mice that refused to run on the wheels during the baseline measurement (travelled a distance <150 m) were excluded from further evaluation [47].

#### 2.6.4. Nesting Behaviour Test

Nesting is an innate behaviour in mice that may be sensitive to pain conditions [48]. Mice were habituated to the nesting cage for 48 h before testing. As nesting material, one 5 × 5 cm^2^ nestlet consisting of pressed virgin cotton was cut into six roughly equal pieces (~1.7 × 2.5 cm^2^). The nest pieces were evenly placed in the four corners and the middle of each long side of the cage (49 × 34 × 16 cm^2^), and the cage space was divided into six equal zones for nesting assessment. The nesting quality score ranged from 0.5 to 6 points and was measured as follows: 0.5 points were assigned to the mouse if it cleared one zone, and 0.5 points were assigned if the mouse shredded the nestlet. The nesting score ranged from 0.5 to 6 points. The nesting behaviour was scored after 120 min of exposure to the initial nesting material [48,49]. A decrease in the nesting score indicates pain-depressed nesting behaviour, suggesting one useful spontaneous nociception behaviour.

#### 2.6.5. Thigmotaxis Behaviour

Thigmotaxis behaviour was evaluated using an open field (40 cm × 30 cm × 18 cm) with a delimited inner zone (12 × 12 cm). Each mouse was transferred to the apparatus and observed for 15 min [46,50]. The number of entries into the inner zone and the total immobility time were analysed by ANY-maze Software (7.0 version, Stoelting Co., Wood Dale, IL, USA). Thigmotaxis behaviour corresponds to a decreased exploration of the inner zone of the open field and indicates anxiety-like behaviour [50].

#### 2.6.6. Forced Swimming Test

Forced swimming is a commonly used assay to study depressive-like behaviour in rodents [51]. The forced swimming test was performed using a cylinder (20 cm diameter and 45 cm height) filled with water (23–25 °C) at a height of 30 cm. Mice were placed in the water, and the time for which the mice remained immobile was quantified in seconds over a period of 2–6 min using a chronometer [46,50]. Immobility was defined as the absence of all movements except those required to maintain the head above water.

#### 2.6.7. Novel Object/Place Recognition Test

To assess cognitive function, we subjected mice to a novel object/place recognition test (NOPRT). NOPRT measures the spatial and working memory of mice using the innate preference of mice for novelty [52,53] and was performed as previously described [52,54]. One day after administration of the last dose of cisplatin, mice were habituated to the test arena (40 cm × 30 cm × 18 cm), with two identical objects placed on the same side of the arena for 5 min (training phase), after which the mouse was returned to its home cage. Thirty minutes later, the mice were transferred back to the arena, which now contained one familiar object placed at the same location as in training and one novel object placed on the opposite end of the arena (testing phase), and they were allowed to explore for another 5 min. Sniffing, climbing, and touching the objects were regarded as exploration behaviour. The exploration time of the familiar and novel object was scored manually. The discrimination index was determined as *[time with the novel object − time with the familiar object]/total exploration time of both objects*.

### 2.7. Evaluation of Other Possible Adverse Effects

#### 2.7.1. Physical and Behavioural Changes

Physical (body weight verified through a scale and hair appearance) and behavioural (irritability, salivation, and tremors) changes were visually evaluated before and throughout the experimental period (during and after cisplatin administration) by the experimenter.

#### 2.7.2. Locomotor Activity

We assessed the effect of cisplatin on the locomotor activity of animals one day after the last cisplatin (2.3 mg/kg, i.p.) or vehicle (control group; 10 mL/kg, i.p.) administration (11th day). The spontaneous locomotor activity was recorded for 15 min in an open-field apparatus (40 cm × 30 cm × 18 cm), and the results of the total distance travelled were obtained by automated analysis ANY-maze™ software (7.0 version, Stoelting Co., Wood Dale, IL, US). The spontaneous locomotor activity was also evaluated on the 30th day after the first cisplatin or vehicle administration by an open-field test [55]. The open-field apparatus consists of a glass box (28 × 18 × 12 cm) divided into nine squares. On the 30th day after the first cisplatin or vehicle administration, each mouse was placed in the apparatus, and the number of squares crossed with all paws and rearing was counted in a 5 min session. The forced locomotor activity was evaluated using the rotarod test. Before the first cisplatin or vehicle dose, all the animals were trained in the rotarod (3.7 cm in diameter, 8 rpm) until they could remain in the apparatus for 60 s without falling. On the 30th day after the first cisplatin (2.3 mg/kg, i.p.) or vehicle (control group; 10 mL/kg, i.p.) dose, the number of falls from the apparatus was recorded for up to 240 s [55].

#### 2.7.3. Biochemical Analysis

The mice received the cisplatin (2.3 mg/kg, i.p.) or vehicle (10 mL/kg, i.p.) administrations. On the 30th day after the first cisplatin dose, they were deeply anaesthetized, and blood was collected by heart puncture. The obtained serum was used for a biochemical assay to assess serum urea nitrogen and serum creatinine levels, as well as the activities of aspartate aminotransferase (AST) and alanine aminotransferase (ALT) enzymes.

### 2.8. Statistical Analysis

Statistical analyses were performed using Graph Pad Prism 8.0 software (Graph Pad, San Diego, CA, USA). Results were expressed as the mean ± standard error of the mean (SEM). The significance of differences between groups was evaluated with a Student’s *t*-test and one-way or two-way analysis of variance (ANOVA) followed by Bonferroni’s post hoc test. To meet the parametric assumptions, the data on the mechanical threshold were log-transformed before analyses. The nesting test scores are reported as medians followed by their 25th and 75th percentiles (interquartile range). The percentages of maximum effect were calculated for the maximal developed responses compared to baseline values or the control group. *p*-values less than 0.05 (*p* < 0.05) were considered statistically significant.

## 3. Results

### 3.1. Cisplatin Induces Prolonged Painful Peripheral Neuropathy in Mice

First, we explored whether the treatment regimen used to induce the peripheral neuropathy model (Figure 1A) causes mechanical allodynia and cold sensitivity. Cisplatin reduced the mechanical PWT of mice from the third cisplatin dose (5th day) until 30 days after the first cisplatin administration, indicating the development of mechanical allodynia (Figure 1B). A PWT reduction of 72 ± 7% was observed on the 11th day after the first cisplatin administration. Mice treated with cisplatin also developed cold sensitivity after the final dose of cisplatin (11th day) compared to the vehicle group (Figure 1C), which remained until the 18th day. Thus, the 11th day after the first cisplatin administration was chosen for subsequent experiments.

**Figure 2:**
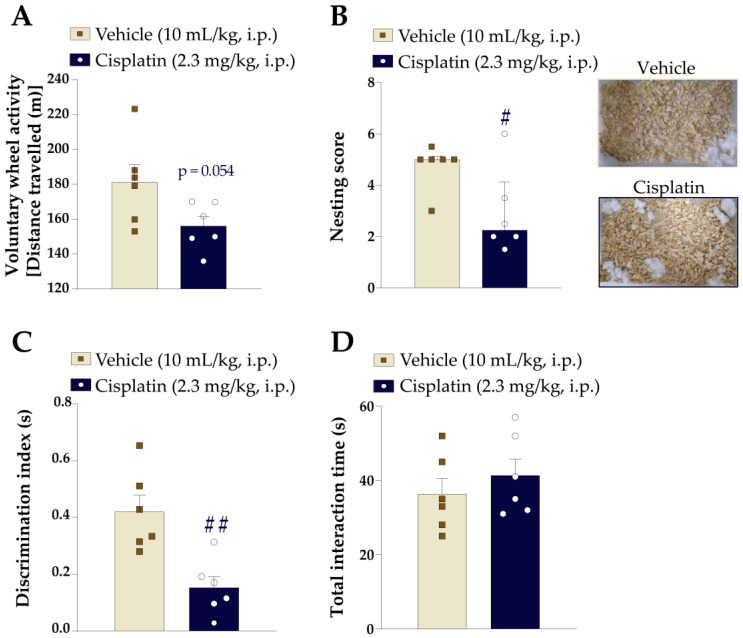
Treatment with cisplatin induced spontaneous nociceptive behaviours and impaired working and spatial memory in mice. Male Swiss mice were treated intraperitoneally (i.p.) with cisplatin (2.3 mg/kg) or its vehicle (10 mL/kg) from day 0 onwards every other day for a total of 10 days to induce the peripheral neuropathy experimental model. On the 11th day, the mice were subjected to behavioural tests. Spontaneous nociception was evaluated by voluntary wheel activity (**A**) and nesting behaviour (**B**). The cognitive function was evaluated by NORPT. The discrimination index demonstrates the preference for the novel object (**C**) and the total interaction time with the novel or familiar object (**D**). The symbols on the bars indicate individual values for each animal. ^#^ *p* < 0.05 and ^##^ *p* < 0.01 vs. vehicle group. Data are expressed as the mean + SEM (n = 6/group) and were analysed by an unpaired two-tailed Student’s *t*-test, except (**B**) nesting score (n = 6/group) data, which are expressed as 25th and 75th percentiles (interquartile range) and were analysed by one-tailed Mann–Whitney test.

Measurements of spontaneous pain were performed one day after the last cisplatin dose. Cisplatin partially decreased the distance travelled in the voluntary activity in the wheel test (Figure 2A) and reduced nesting behaviour compared to the vehicle group (Figure 2B). Mice treated with cisplatin showed a decreased preference for the novel object when compared to vehicle-treated mice, indicating impairment in cognitive function (Figure 2C). No differences were observed in the total time the animals interacted with objects, indicating that the decreased discrimination index of cisplatin-treated mice was not due to reduced interest (Figure 2D). Moreover, cisplatin did not cause anxiety and depressive-like behaviour, as evaluated by the number of entries to the inner zone and total immobility time in the open-field apparatus, and it did not increase the immobility time in the forced swimming test (Table S1).

Furthermore, cisplatin neither changed the body weight (Table S2) nor caused behavioural alterations (e.g., irritability, salivation, or tremors) compared with the vehicle group. Cisplatin treatment also did not affect the locomotor function of mice, as demonstrated by the total distance travelled (m) in the open-field apparatus on the 11th day (Table S2). In addition, cisplatin treatment affected neither the mice’s spontaneous locomotor function evaluated on the 30th day, as demonstrated by crossing and rearing numbers (Table S2), nor the forced locomotor activity, as shown by the mice’s number of falls in the rotarod test (Table S2). The treatment with cisplatin that induced peripheral neuropathy did not cause changes in the urea and creatinine levels or ALT and AST enzyme activities (Table S3).

### 3.2. Kinin B_1_ and B_2_ Receptors Contribute to Mechanical Allodynia in Cisplatin-Induced Peripheral Neuropathy in Mice

**Figure 3:**
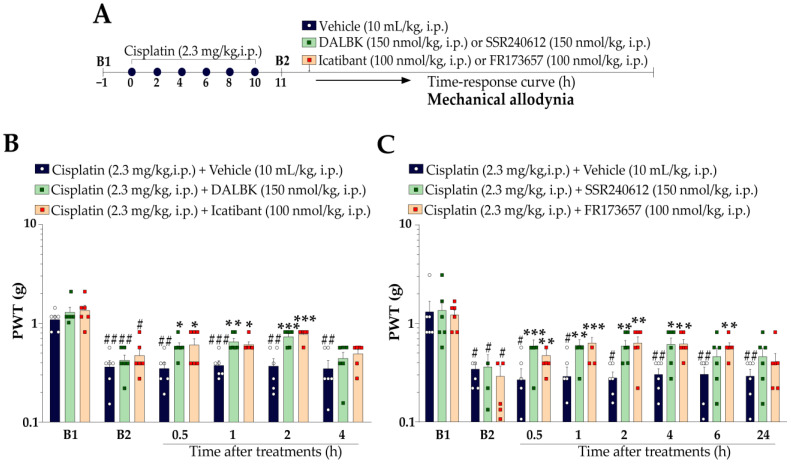
Therapeutic effect of kinin B_1_ and B_2_ receptor antagonists on cisplatin-induced mechanical allodynia. (**A**) *Therapeutic protocol*: Male Swiss mice were treated intraperitoneally (i.p.) with cisplatin (2.3 mg/kg) every 48 h for 10 days. On the 11th day after the first cisplatin dose, the animals received a single administration of DALBK or SSR240612 (150 nmol/kg, i.p., peptide and non-peptide kinin B_1_ receptor antagonist, respectively), Icatibant or FR173657 (100 nmol/kg, i.p., peptide and non-peptide kinin B_2_ receptor antagonist, respectively), or vehicle (10 mL/kg, i.p.). Time–response curve for mechanical allodynia after treatment with DALBK or Icatibant (**B**) and SSR240612 or FR173657 (**C**). Baseline 1 (B1) values were measured before the first cisplatin dose. Baseline 2 (B2) values were measured on the 11th day after the first cisplatin dose and before the treatments. The symbols on the bars indicate individual values for each animal. ^#^ *p* < 0.05, ^##^ *p* < 0.01 and ^###^ *p* < 0.001 vs. B1 values. * *p* < 0.05, ** *p* < 0.01, and *** *p* < 0.001 vs. cisplatin plus vehicle group. Data are expressed as the mean + SEM (n = 6/group) and were analysed by two-way ANOVA followed by the Bonferroni post hoc test. PWT: paw withdrawal threshold.

First, utilizing a therapeutic protocol, we evaluated whether pharmacological blockade using the kinin B_1_ and B_2_ receptor antagonists reduces the cisplatin-induced mechanical allodynia (Figure 3A). The peptide antagonists for kinin B_1_ (DALBK, 150 nmol/kg, i.p.) and B_2_ (Icatibant, 100 nmol/kg, i.p.) receptors reduced cisplatin-induced mechanical allodynia from 0.5 up to 2 h after their administration (Figure 3B), with reductions of 52 ± 7% and 57 ± 5% at 2 h after treatments, respectively. Similar effects were observed for non-peptide kinin B_1_ and B_2_ receptor antagonists. Non-peptide antagonists for kinin B_1_ (SSR240612, 150 nmol/kg, i.p.) and B_2_ (FR173657, 100 nmol/kg, i.p.) receptors decreased the cisplatin-induced mechanical allodynia from 0.5 up to 4 h (reduction of 31 ± 8% at 0.5 h) and from 0.5 up to 6 h (reduction of 34 ± 9% at 2 h) after their treatments (Figure 3C), respectively. Peptide and non-peptide antagonists for kinin B_1_ or B_2_ receptors failed to reduce cisplatin-induced cold sensitivity.

**Figure 4:**
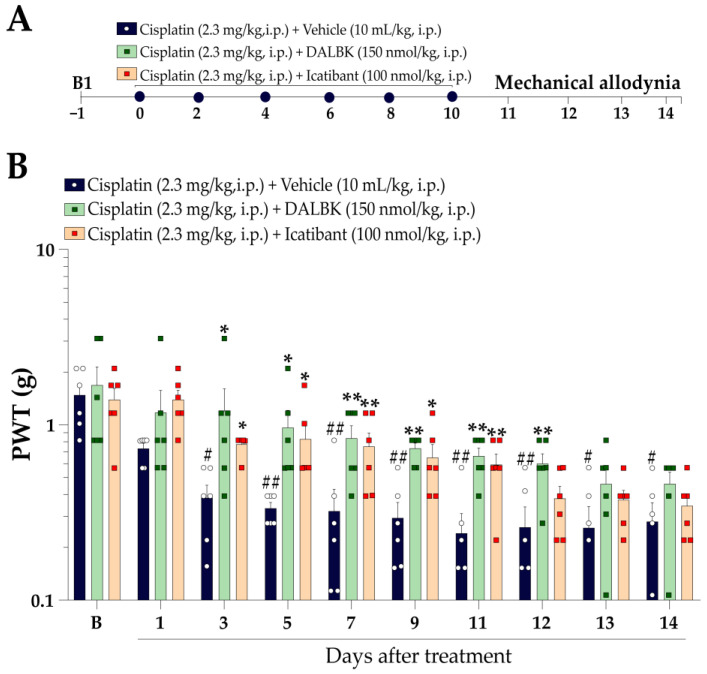
Preventive effect of kinin B_1_ and B_2_ receptor antagonists on cisplatin-induced mechanical allodynia. (**A**) *Preventive protocol*: Male Swiss mice were treated concomitantly by intraperitoneal (i.p.) injections with cisplatin (2.3 mg/kg) plus peptide kinin B_1_ (DALBK, 150 nmol/kg, i.p.) or B_2_ (Icatibant, 100 nmol/kg, i.p.) receptor antagonists or vehicle (10 mL/kg, i.p.) every 48 h for 10 days. Time–response curve for PWT throughout the treatment with cisplatin plus DALBK or Icatibant (**B**). Baseline (B) values were measured before the first cisplatin dose. The symbols on the bars indicate individual values for each animal. ^#^ *p* < 0.05 and ^##^ *p* < 0.01 vs. B1 values. * *p* < 0.05 and ** *p* < 0.01 vs. cisplatin plus vehicle group. Data are expressed as the mean + SEM (n = 6/group) and were analysed by two-way ANOVA followed by the Bonferroni post hoc test. PWT: paw withdrawal threshold.

Next, we assessed the effect of peptide antagonists for kinin B_1_ and B_2_ receptors in preventing the mechanical allodynia development induced by cisplatin (Figure 4A). DALBK (150 nmol/kg, i.p.) effectively prevented mechanical allodynia development when administered concomitantly with cisplatin until the 12th day, with prevention of 55 ± 21% on the 5th day after the first treatment. Similarly, Icatibant (100 nmol/kg, i.p.) also prevented mechanical allodynia development when administered concomitantly with cisplatin until the 11th day after the first treatment, with prevention of 43 ± 16% on the 5th day (Figure 4B). Peptide kinin B_1_ or B_2_ receptor antagonists did not prevent cisplatin-induced cold sensitivity.

### 3.3. Kinin B_1_ and B_2_ Receptor Agonists Enhanced Cisplatin-Induced Mechanical Nociception, Which Was Reversed by B_1_ and B_2_ Receptor Antagonists

**Figure 5:**
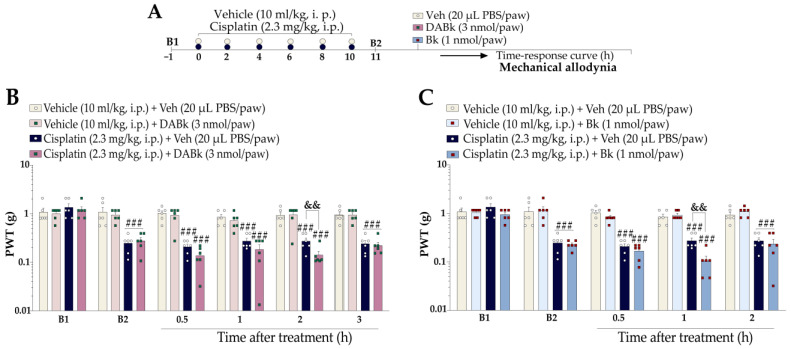
Sub-nociceptive doses of B_1_ and B_2_ receptor agonists intensified cisplatin-induced mechanical nociception. (**A**) Male Swiss mice were treated intraperitoneally (i.p.) with cisplatin (2.3 mg/kg) every 48 h for 10 days. On the 11th day after the first cisplatin injection, the animals were treated with sub-nociceptive doses of agonists kinin B_1_ (DABk; 3 nmol/paw, i.pl.) or B_2_ (Bk; 1 nmol/paw, i.pl.) receptor or vehicle (20 μL PBS/paw, intraplantar, i.pl.) via the intraplantar route. PWT (**B**,**C**) was assessed from 0.5 h up to 3 h after injection of sub-nociceptive doses of the agonists. Baseline 1 (B1) values were measured before cisplatin or vehicle administration. Baseline 2 (B2) values were measured on the 11th day after the first cisplatin dose and before the treatments. The symbols on the bars indicate individual values for each animal. ^###^ *p* < 0.001 vs. Veh plus Veh group. ^&&^ *p* < 0.01 vs. cisplatin plus Veh. Data are expressed as the mean + SEM (n = 6/group) and were analysed by two-way ANOVA followed by the Bonferroni post hoc test, except for the DABk effect on mechanical allodynia enhanced (Student’s *t*-test). Veh: vehicle; PBS: phosphate-buffered saline; PWT: paw withdrawal threshold.

First, we evaluated whether low doses of kinin B_1_ and B_2_ receptor agonists could enhance cisplatin-induced nociceptive behaviour (Figure 5A). Intraplantar (i.pl.) DABk (3 nmol/paw; a sub-nociceptive dose of kinin B_1_ receptor agonist) enhanced cisplatin-induced mechanical nociception 2 h after its administration when compared to the cisplatin plus vehicle group (Figure 5B). Likewise, i.pl. Bk (1 nmol/paw; a sub-nociceptive dose of kinin B_2_ receptor agonist) enhanced cisplatin-induced mechanical nociception 1 h after its administration when compared to the cisplatin plus vehicle group (Figure 5C). As expected, sub-nociceptive doses of the Bk and DABk agonists did not alter the mechanical sensitivity in animals previously treated with vehicle (Figure 5B,C).

**Figure 6:**
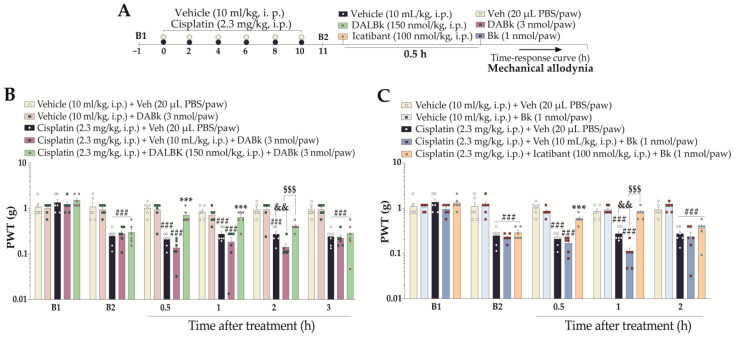
Kinin B_1_ and B_2_ receptor antagonists prevent the enhancement of B_1_ and B_2_ receptor agonist-induced mechanical nociception in cisplatin-treated mice. (**A**) Male Swiss mice were treated intraperitoneally (i.p.) with cisplatin (2.3 mg/kg) every 48 h for 10 days. On the 11th day after the first cisplatin injection, the animals received a single administration of DALBK (150 nmol/kg, i.p.), Icatibant (100 nmol/kg, i.p.), or vehicle (10 mL/kg, intraperitoneal, i.p.). After 0.5 h, sub-nociceptive doses of the respective agonists, i.e., DABk (3 nmol/paw, i.pl.) or Bk (1 nmol/paw, i.pl.), or vehicle (20 μL PBS/paw, intraplantar, i.pl.) were administered via the intraplantar route. PWT (**B**,**C**) was assessed from 0.5 h up to 3 h after the treatments. Baseline 1 (B1) values were measured before cisplatin or vehicle administration. Baseline 2 (B2) values were measured on the 11th day after the first cisplatin dose and before the treatments. The symbols on the bars indicate individual values for each animal. ^###^ *p* < 0.001 vs. Veh plus Veh group. ^&&^ *p* < 0.01 vs. cisplatin plus Veh. *** *p* < 0.001 vs. cisplatin plus Veh. ^$$$^ *p* < 0.001 vs. cisplatin plus DABk/Bk group. Data are expressed as the mean + SEM (n = 6/group) and were analysed by two-way ANOVA followed by the Bonferroni post hoc test, except for the DABk effect on mechanical allodynia (Student’s *t*-test). Veh: vehicle; PBS: phosphate-buffered saline; PWT: paw withdrawal threshold.

Since low doses of kinin B_1_ and B_2_ receptor agonists intensified the cisplatin-induced mechanical nociception, we evaluated whether kinin receptor antagonists prevented this behaviour (Figure 6A). The kinin B_1_ receptor antagonist DALBK (150 nmol/kg, i.p., 0.5 h prior agonist injection) prevented the enhancement of mechanical nociception, with an effect of 31 ± 4 % 2 h after its administration (Figure 6B). Icatibant (100 nmol/kg, i.p., 0.5 h prior to agonist injection), a kinin B_2_ receptor antagonist, markedly prevented the enhancement of mechanical nociception, with an effect of 75 ± 11% at 1 h after its administration (Figure 6C).

### 3.4. Antisense Oligonucleotides for Kinin B_1_ or B_2_ Receptors Attenuated the Cisplatin-Induced Mechanical Allodynia

**Figure 7:**
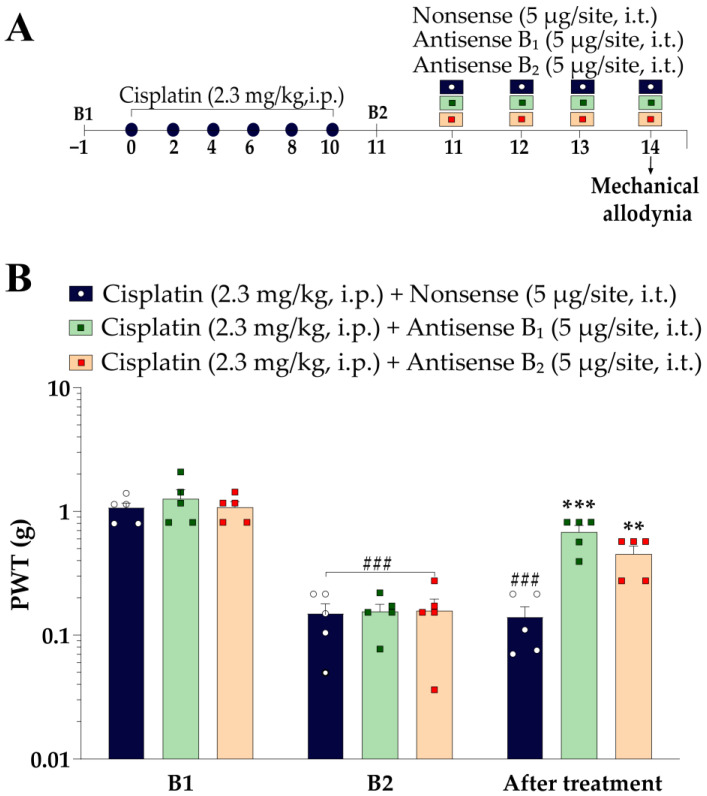
Antisense oligonucleotides for kinin B_1_ and B_2_ receptors relieved cisplatin-caused mechanical allodynia. (**A**) Male Swiss mice were treated by intraperitoneal (i.p.) injections with cisplatin (2.3 mg/kg) every 48 h for 10 days. Mice were treated with antisense oligonucleotides targeting kinin B_1_ and B_2_ receptors, and nonsense control was administered via the intrathecal (i.t.) route for three consecutive days every 12 h and 1 h before assessment of mechanical allodynia on the 14th day after induction of peripheral neuropathy by cisplatin. (**B**) Mechanical allodynia was evaluated on the 14th day after cisplatin-induced peripheral neuropathy. B1 values were measured before the first cisplatin dose. B2 values were measured on the 11th day after the first cisplatin dose and before the treatments. Results are presented as mean + SEM (n = 5–6/group). The symbols on the bars indicate individual values for each animal. ^###^ *p* < 0.001 compared to baseline threshold (B1). ** *p* < 0.01; *** *p* < 0.001 compared to the nonsense group. Two-way ANOVA repeated measures followed by Bonferroni’s post hoc test. PWT: paw withdrawal threshold.

To reinforce the involvement of kinin B_1_ and B_2_ receptors in cisplatin-induced mechanical allodynia, we silenced the gene expression of the kinin B_1_ or B_2_ receptor using antisense oligonucleotides (Figure 7A). Antisense oligonucleotides for kinin B_1_ and B_2_ receptors attenuated cisplatin-induced mechanical allodynia, with inhibition of 57 ± 8% and 33 ± 7%, respectively (Figure 7B). On the other hand, control oligonucleotide injection (nonsense) did not affect the cisplatin-induced mechanical allodynia. Antisense oligonucleotides did not attenuate cisplatin-induced cold sensitivity.

## 4. Discussion

Peripheral neuropathy is one of the most common adverse effects of platinum-based chemotherapy drugs such as cisplatin. CIPN considerably impacts cancer treatment strategies, leading to a dose reduction or treatment discontinuation and negatively affecting the patients’ quality of life [9,11,56]. The increasing number of cancer survivors and the lack of treatment to prevent or manage CIPN emphasizes the urgent need to unveil the pathophysiological mechanisms of CIPN to develop effective therapeutic strategies. This study provided the first evidence of the involvement of the kinin B_1_ and B_2_ receptors in cisplatin-induced painful peripheral neuropathy using pharmacological and genetic tools. Therefore, kinin receptors seem crucial to mediating mechanical nociception in cisplatin-induced peripheral neuropathy, suggesting that these receptors may also be critical in a clinical setting. Moreover, we demonstrated that kinin B_1_ and B_2_ receptor antagonists have therapeutic potential to relieve cisplatin-associated pain symptoms.

Clinically, cisplatin dose is a determinant for peripheral neuropathy development [11,12,57,58]. CIPN commonly manifests as an increased perception of innocuous (allodynia) or noxious (hyperalgesia) stimuli, which are hallmark symptoms of neuropathic pain [6,8,56]. In the present study, cisplatin treatment resulted in prominent and persistent mechanical allodynia lasting at least 30 days. This result agrees with previous data demonstrating the development of mechanical allodynia after the third cisplatin dose in a different strain of mice [41,42,59].

Changes in peripheral sensory sensations concerning cold stimuli are commonly associated with neuronal toxicity caused by antineoplastic agents such as platinum-based agents [11,12,56]. Here, we observed that six doses of cisplatin increased cold sensitivity. Although our results are consistent with previous studies [60,61,62], the changes in thermal hypersensitivity (cold and heat) caused by cisplatin are controversial [56] and seem to be more associated with oxaliplatin use—another platinum-based agent. Since literature data demonstrate no differences in the onset or severity of CIPN between male and female mice, we evaluated the cisplatin effects only on male but not female mice [56,63,64,65].

In this study, we demonstrated that cisplatin treatment induced spontaneous pain-like behaviours. The reduction in the spontaneous wheel-running activity and nesting performance reflects depressed pain behaviours typical during painful conditions in rodents [47,48,66]. Reductions in these behaviours were previously described for different pain models [47,49,67]. Nonetheless, these are the first data showing the effects of cisplatin on nest building and wheel running, indicating spontaneous pain development, that is, pain in the absence of a stimulus.

In addition to painful symptoms observed in CIPN, after undergoing cancer chemotherapy, patients also present a high risk of cognitive impairment—another neurotoxic condition of chemotherapy agents [53,68,69]. Chemotherapy-induced cognitive impairment, commonly known as *chemobrain*, consists of damage in several cognitive domains, including impairment in working memory, attention, processing speed, concentration, and executive function [54,68]. Cognitive dysfunction is also related to cisplatin treatment, as it crosses the blood–brain barrier in low concentrations [52,70]. In the present study, repeated cisplatin treatment caused a decreased preference for the novel object, indicating impaired working memory and spatial recognition [54]. These results corroborate previous data showing that cisplatin induces cognitive impairment [52,53,54].

Pain and humour disorders, such as depression, may develop secondarily to each other or may coexist. In general, depression may cause increased pain perception by patients who may be more likely to develop chronic pain [71]. Cancer patients undergoing chemotherapy treatment present symptoms of depression and anxiety, in addition to neuropathic pain symptoms [72,73]. In our study, cisplatin caused neuropathic pain symptoms but not depressive- and anxiety-like behaviours since it altered neither the mice’s immobility time in the forced swimming test nor thigmotaxis behaviour, unlike in other studies [74,75,76]. These discrepancies between the results may be due to the different administration schedules, doses of cisplatin, or differences between the animal strains tested [74,75,76]. Thus, it is important to better elucidate such conditions underlying chemotherapeutic treatment, as well as cognitive impairment and mood disorders, in experimental models, since they can influence or be influenced by chronic pain states [71,77].

The cisplatin dose used in this study promoted nociceptive responses without causing damage to the general health of the mice. On the other hand, higher doses of cisplatin result in weight loss accentuated after the second cisplatin administration [78]. Furthermore, cisplatin did not cause motor impairments, as evaluated by spontaneous and forced locomotor activity.

The pathological mechanisms underlying CIPN development have been widely debated [9,11,64,65]. Potential targets that might be involved in cisplatin-induced painful peripheral neuropathy pathophysiology are the kinin B_1_ and B_2_ receptors, which are involved in various painful conditions, including those induced by other chemotherapy drugs [27,31,32,33,39].

Kinins (bradykinin and kallidin), as well as their active metabolites (des-Arg^9^-Bk and des-Arg^10^-kallidin), are endogenous peptides that mediate inflammatory and painful processes via the kinin B_1_ and B_2_ receptors, respectively [79,80]. In the present study, pharmacological antagonism and gene silencing using antisense oligonucleotides for the kinin receptors attenuated cisplatin-induced mechanical allodynia. These findings indicate that cisplatin can promote the painful symptom characteristic of CIPN in male mice in a kinin B_1_ and B_2_ receptor-dependent manner. Since no study has shown discrepant kinin receptor effects on painful conditions in male and female experimental animals, we evaluated the antinociceptive effect of kinin B_1_ and B_2_ receptor antagonists only in male mice.

The systemic administration of peptide kinin B_1_ and B_2_ antagonists decreased mechanical allodynia in the therapeutic and preventive protocol. It is worth mentioning that nociceptive tests in the preventive protocol were carried out 24 h post administration of the antagonists, indicating a lasting effect of the peptide kinin B_1_ and B_2_ antagonists once efficacy was reached. In the therapeutic protocol, non-peptide kinin B_1_ and B_2_ receptor antagonists SSR240612 e FR173657 exerted a more prolonged antiallodynic effect than the peptide antagonists. Similarly, antinociceptive effects more prolonged from non-peptide antagonists than peptide antagonists were evidenced in a fibromyalgia model and paclitaxel-induced pain syndrome [23,27]. Notwithstanding the longer-lasting effect observed for non-peptide antagonists, the inhibition percentage of mechanical allodynia was similar to that caused by peptide antagonists. Therefore, their effects were not evaluated in the preventive protocol.

The ability of kinin antagonists to attenuate cisplatin-induced mechanical allodynia can be explained by the constitutive expression of kinin B_1_ and B_2_ receptors in structures important for nociceptive transmission, such as nociceptive neurons, the dorsal root ganglion, and spinal cord [25,26,28,81]. Still, immune cells, such as monocytes, neutrophils, and microglia, also express kinin receptors [80,82]. In this sense, microglia activation on the spinal cord was previously demonstrated in the cisplatin-induced peripheral neuropathy model [83].

Therefore, our results agree with previous studies that have linked kinin receptors to the pathogenesis of different acute and chronic pain models, highlighting the role of these receptors in pain hypersensitivity following mechanical stimulus [18,19,21,22,23,84]. In particular, kinin receptors also contribute to mechanical hypersensitivity induced by chemotherapeutic agents such as paclitaxel and vincristine [27,31,33].

To contribute to our hypothesis that kinin B_1_ and B_2_ receptors mediate cisplatin-induced painful symptoms, mice previously treated with cisplatin received sub-nociceptive doses of kinin B_1_ and B_2_ receptor agonists. Local exposure to agonists of kinin receptors (at doses that generally do not cause nociception) is associated with more prolonged and intensified nociceptive behaviours [18,23]. Kinin B_1_ and B_2_ receptor agonists—DABK and Bk, respectively—enhanced cisplatin-induced mechanical nociception. Similarly, chronic pain studies have reported hypersensitivity to sub-nociceptive doses of kinin B_1_ and B_2_ agonists [18,23]. The respective antagonist reduced this increased nociceptive response, providing additional evidence of the involvement of kinin B_1_ and B_2_ receptors in cisplatin-induced pain hypersensitivity.

The intrathecal administration of antisense oligonucleotides targeting kinin B_1_ and B_2_ receptors decreased cisplatin-induced mechanical allodynia. Our results are consistent with previous studies showing that genetic deletion of kinin receptors effectively reduces pain responses in different experimental models [18,85]. As mentioned before, in addition to their expression at the peripheral level, the kinin B_1_ and B_2_ receptors also are found or upregulated in the spinal cord, astrocytes, and microglia in the central nervous system, contributing to chronic pain states such as neuropathic pain [82,86,87]. This explains the ability of intrathecal antisense oligonucleotides targeting kinin B_1_ and B_2_ receptors to attenuate cisplatin-induced mechanical allodynia.

Although kinin receptor antagonists and antisense oligonucleotides reduced cisplatin-induced mechanical allodynia, in our study, they did not reduce cold hypersensitivity. These results are in agreement with a study by Gonçalves et al. (2021), which disregards the involvement of kinin receptors in cold hypersensitivity. Unlike our findings, kinin receptor antagonists attenuated the cold sensitivity in a spinal nerve ligation and fibromyalgia model [22,23]. However, considering that TRPA1 is a harmful cold sensor and that the activation of B_1_ and B_2_ receptors causes sensitization of TRP channels, including TRPA1 [25,39,88,89,90,91,92], kinin receptors may be indirectly involved in cold hypersensitivity. Therefore, it is essential to better elucidate the mechanisms of cold allodynia in the cisplatin-induced neuropathy model and define the role of kinin receptors in this condition.

In addition to neurotoxicity, cisplatin is also associated with nephrotoxic effects [6,8]. In this regard, cisplatin did not alter the urea and creatinine levels of animals in a previous study [93]. Interestingly, both kinin B_1_ and B_2_ receptors seem to be involved in cisplatin-induced nephrotoxicity once the deletion and blockage of kinin B_1_ and B_2_ receptors have been shown to protect against cisplatin-induced acute kidney injury [34,35]. Furthermore, Estrela et al. (2017) showed that the deletion and blockage of the kinin B_1_ receptor prevented the downregulation of organic transporters in kidney cisplatin-induced toxicity, increasing the cisplatin efflux and consequently protecting against cisplatin nephrotoxicity [94]. Thus, using kinin receptor antagonists to relieve painful symptoms could also help to protect against cisplatin-induced renal toxicity. Kinin antagonists might also present additional beneficial effects, such as avoiding cancer cell proliferation since kinin antagonists alone or in association with chemotherapeutics, including cisplatin, inhibit the growth of ovarian and lung tumour cells [95,96,97]. In general, an ideal therapeutic strategy should act synergistically, aiding in antitumour action and protecting against neurotoxic and nephrotoxic effects. Thus, our study and previous studies support the potential of kinin antagonists in these conditions induced by cisplatin and a possible synergistic effect on antitumour activity.

Our findings show that the mechanisms mediated by kinin B_1_ and B_2_ receptors contribute to cisplatin-induced peripheral neuropathy symptoms, especially in mechanical nociception, indicating that kinin B_1_ and B_2_ receptors are potential pharmacological targets to relieve the pain symptoms associated with cisplatin. Furthermore, considering the safety and tolerability of the kinin B_2_ receptor antagonist, Icatibant, which has already been approved for treatment of hereditary angioedema [98], our data suggest a possible clinical repositioning of Icatibant for patients with cancer undergoing chemotherapy with cisplatin. Therefore, regulating the activation of kinin B_1_ and B_2_ receptors is a promising alternative to avoid dose reduction or interruption of chemotherapy treatment, in addition to contributing to the treatment of painful symptoms of cancer survivors and re-establishing their quality of life.

## Data Availability

Not applicable.

## Supplementary Materials

The following supporting information can be downloaded at: https://www.mdpi.com/article/10.3390/pharmaceutics15030852/s1, Table S1: Anxiety and depressive-like behaviours after cisplatin or vehicle injections. Table S2: Body weight and locomotor activity evaluations after cisplatin or vehicle injections. Table S3. Biochemical analysis after cisplatin or vehicle injections.
