# Supplementary material for: Kinin B1 and B2 Receptors Contribute to Cisplatin-Induced Painful Peripheral Neuropathy in Male Mice
Source: Pharmaceutics. 2023 Mar 6;15(3):852. doi: 10.3390/pharmaceutics15030852 (PMC10051506; doi:10.3390/pharmaceutics15030852)
Supplement: Supplementary file 1 [file pharmaceutics-15-00852-s001.zip › pharmaceutics-2184484-supplementary.pdf]

pharmaceutics

Supplementary Materials: Kinin B_1 and B_2 Receptors Contribute to Cisplatin-Induced Painful Peripheral Neuropathy in Male Mice

Gabriela Becker, Maria Fernanda Pessano Fialho, Indiara Brusco and Sara Marchesan Oliveira

Table S1. Anxiety and depressive-like behaviours after cisplatin or vehicle injections.

Treatments	Forced swimming test [(Immobility time (s)]	Thigmotaxis behaviour (Number of entries into the inner zone)	Thigmotaxis behaviour [Total immobility time(s)]
**Vehicle** (10 ml/kg. i.p.)	67.66 ± 2.90	230.0 ± 10.0	133.7 ± 18.04
**Cisplatin** (2.3 mg/kg. i.p.)	81 ± 13.30	200.41 ± 18.67	161.86 ± 34.82

Mice were treated with vehicle (10 mL/kg. i.p.) or cisplatin (2.3 mg/kg. i.p.). On the 11th day after the first cisplatin or vehicle dose, the animals were subjected to forced swimming test and thigmotaxis behaviour in the open-field apparatus. All parameters were assessed with 6 mice per group (n=6/group). Data were expressed as the mean ± SEM and analysed by Student's t test.

Table S2. Body weight and locomotor activities evaluations after cisplatin or vehicle injections.

Treatments	Body weight (g)				Locomotor activity			
	Before administration	9th day	15th day	21th day	Total travelled distance (m)	Crossing number	Rearing number	Number of falls
**Vehicle** (10 ml/kg. i.p.)	27 ± 1	31 ± 0.91	32.5 ± 0.67	32.83 ± 0.47	219 ± 11	123 ± 3	66 ± 5	1.16 ± 0.47
**Cisplatin** (2.3 mg/kg. i.p.)	28 ± 0.66	30 ± 0.25	29.66 ± 0.80	31.5 ± 1.2	187 ± 11	110 ± 8	63 ± 4	1.16 ± 0.6

Mice were treated with vehicle (10 mL/kg. i.p.) or cisplatin (2.3 mg/kg. i.p.). The body weight was evaluated before, during, and after the cisplatin administration. On the 11th day after the first cisplatin or vehicle dose, the animals were subjected to locomotor activity evaluations in the open-field apparatus and on the 30th day, they were also tested for spontaneous and forced locomotor activities. All parameters were assessed with 6 mice per group (n=6/group). Data were expressed as the mean ± SEM and analysed by Student's t test.

Table S3. Biochemical analysis after cisplatin or vehicle injections.

Treatments	Creatinine (mg/dL)	Urea (mg/dL)	ALT (U/L)	AST (U/L)
**Vehicle** (10 ml/kg. i.p.)	0.779 ± 0.06	42.027 ± 3.17	51.070 ± 8.83	11.290 ± 2.69
**Cisplatin** (2.3 mg/kg. i.p.)	0.736 ±0.07	52.288 ± 5.95	41.5257 ± 5.48	11.872 ± 4.07

Mice were treated with vehicle (10 mL/kg. i.p.) or cisplatin (2.3 mg/kg. i.p.). On the 30th day after the first cisplatin administration, they were deeply anaesthetized, and blood was collected. The serum obtained was used for biochemical assay to assess serum urea nitrogen and serum creatinine levels and the activities of aspartate aminotransferase (AST) and alanine aminotransferase (ALT) enzymes. Data were expressed as the mean ± SEM (n=6/group) and analysed by Student's t test.
